# Health risk assessment and trace element analysis in tobacco products and user blood samples from urban and rural Karachi

**DOI:** 10.1038/s41598-025-16123-0

**Published:** 2025-10-01

**Authors:** Nusrat Begum Jalbani, Shakeel Haider Solangi, Shahid Bhutto, Ho Soon Min, Humaira Khan, Hafiz Rub Nawaz, Maira Bhatti, Saima Imad

**Affiliations:** 1 PCSIR Laboratories, Complex, Karachi, 75280 Pakistan; 2 DR. M.A. KAZI Institute of Chemistry, University of Sindh, Jamshoro, Sindh 76080 Pakistan; 3 Faculty of Health and Life Sciences, INTI International University, Persiaran Perdana BBN, Putra Nilai, Negeri Sembilan, Nilai, 71800 Malaysia

**Keywords:** Metals in blood, Health policy, Residents exposure, Target hazard quotient, Hazard index, Carcigenic risk, Environmental chemistry, Analytical chemistry, Environmental sciences, Chemistry

## Abstract

Cigarette smoking and use of smokeless tobacco products (STPs) such as gutka, mainpuri, and naswar are major public health concerns in Pakistan. This study assessed toxic trace elements (TTEs) Cadmium (Cd), Manganese (Mn), Lead (Pb), and Zinc (Zn) in the blood of male and female tobacco users and non-users from urban and rural Karachi, along with associated health risks. A total of 190 blood and 120 tobacco samples (30 cigarettes, 90 STPs) were analyzed. Cd, Mn, and Pb were measured using electrothermal atomic absorption spectrometry (ETAAS), and Zn by flame atomic absorption spectrometry (FAAS) after acid digestion. The study revealed significantly higher metal concentrations in urban users due to combined exposure from tobacco and environmental pollution. Cd levels in gutka (1.11 mg/kg) and cigarettes (1.035 mg/kg) exceeded WHO’s limit (0.5 mg/kg). Urban male smokers had the highest blood Pb (0.108 ± 0.01 mg/L), over tenfold above the WHO limit, while urban females showed the highest Mn (0.031 mg/L). Rural male mainpuri users had the highest Zn level (2.76 ± 0.16 mg/L). Cd in cigarette posed the highest cancer risk (CR: 9.14 × 10⁻⁴); gutka users had a CR of 6.74 × 10⁻⁴. Female gutka users had the highest Pb intake (0.513 mg/kg/day). The findings call for urgent biomonitoring, regulation, and public health interventions.

## Introduction

Cigarette and smokeless tobacco products (STPs) like gutka, mainpuri, and naswar are widely used in Pakistan, especially among youth often resulting in long-term addiction^1^. Their affordability, flavor, and availability contribute to rising addiction rates^2^. Tobacco use, linked to over 8 million global deaths annually^3,4^remains a major public health concern in Pakistan, affecting more than 25.4 million people^5^including women and youth^6^. Although smoking is legally restricted in Pakistan to designated public areas. According to the Pakistan Demographic and Health Survey, 46% of men and 5.7% of women use tobacco^7^. In Karachi, STPs often contain harmful additives, antibiotics^8^and toxic metals like Pb and Cd, frequently exceeding WHO/FAO recommended safety limits^9^.

Human exposure to trace toxic elements (TTEs) like Cd, Pb, Mn, and Zn is an increasing public health concern in Karachi, home to over 20.3 million people. These elements originate from industrial emissions, soil, vehicle pollution, tobacco use, and environmental contamination^10^. Smokeless tobacco products like gutka, mainpuri, and naswar are significant sources of toxic and carcinogenic compounds, containing addictive substances and TTEs that can accumulate in the body even at low levels^11,12^. Trace toxic elements can enter the body through inhalation or ingestion, accumulating in vital organs such as the brain, lungs, liver, and kidneys, leading to toxic effects^13,14^. Chronic exposure is linked to cardiovascular disease, COPD, and approximately 90% of lung cancer cases^15^. While Zn is biologically essential, excessive exposure from mining, industry, agriculture, and wastewater discharge can lead to nausea, headaches, immune dysfunction, and mitochondrial damage^16,17^. Recognizing these risks is vital for effective public health policies and regulatory measures^18^.

Blood is the primary biological matrix for assessing the accumulation of potentially TTEs in the human body. However, due to the invasive nature, pain, and logistical challenges of blood collection including issues with transport, storage, and ethical considerations alternative biomarkers are being explored^19^. Sample preparation can be complex and time-consuming, with several digestion techniques reported, including microwave-assisted acid digestion^20^ultrasound-assisted dispersive solid-phase extraction, dry and wet ashing^21^and conventional wet acid digestion^22^. Among these, microwave-assisted and conventional wet acid digestion are commonly used for the pre-concentration of Cd and Pb in food, water, and biological samples^23^. This study aims to determine the levels of Cd, Mn, Pb, and Zn in whole blood samples from 190 individuals aged 60 years approx., including male and female smokers, users of STPs, and non-users from both urban and rural Karachi. Additionally, the study evaluates these elements in cigarette and STPs samples. The study conducted in Karachi, a major industrial city with high environmental pollution from vehicular and industrial emissions and coal combustion^24^. The research also calculates Target Hazard Quotient (THQ), Hazard Index (HI), and Carcinogenic Risk (CR) to assess potential health risks from tobacco-related metal exposure.

## Materials and methods

### Reagents and chemicals

Nitric acid (HNO_3_, 65%), hydrogen peroxide (H_2_O_2_, 35%), and other reagents were used of analytical-grade and purchased from E. Merck (Darmstadt, Germany). Ultrapure water was utilized throughout the experimental work. Stock standard solutions for Cd, Mn, Pb and Zn (1000 mg/L) were procured from Merck (Darmstadt, Germany) and traceable to the National Institute of Standards and Technology (NIST). Standard Reference Material 1575a for TTEs in Pine Needles (Pinus taeda) were purchased from NIST, USA. Working standards for the corresponding metals were freshly prepared daily by diluting the stock solutions with Ultrapure water. Validation of proposed methodology was ensured through the SRM and spiking in current samples as recovery tests.

### Instrumentation

A Hitachi Model Z-8000 electro-thermal/flame Atomic Absorption Spectrometry (ET-AAS/FAAS) was used to determine the levels of Cd, Pb, Mn and Zn in cigarette and STPs (gutka, mainpuri, and naswar) samples, whole blood samples from male and female smokers and users of STPs including non-users/control blood samples. The concentrations of Cd, Mn, and Pb were determined using ET-AAS, while -FAAS was employed for Zn determination^25^. Calibration standard curves, SRMs, and spiking recovery tests were performed for each metal. To evaluate accuracy and precision, multiple replicates (typically 2–3 per sample) were analyzed for cigarette, STPs and each blood sample to ensure reliable results.

### Cigarette and STPs samples collection, preservation, and handling

About 10 brands of Cigarette and STPs (Gutka, Mainpuri and Naswar) in triplicate were purchased from local retail shops in Karachi in triplicate. There were 120 number of total samples. These samples were marked and packed in polythene bags. The samples were kept in freezer in the laboratory until starting sample preparation.

### Blood sample collection, preservation, and handling

A total of 190 venous blood samples were collected from urban and rural areas of Karachi between 2023 and 2024, following strict safety protocols. The study was approved by the Institutional Review Committee of PCSIR (Approval No. PCSIR/IRC/2023/027), and informed consent was obtained from all participants to ensure confidentiality and ethical compliance.

Participants were divided into two groups like Group 1 (140 individuals): Male and female users of cigarettes (10–20 g/day) and STPs (Gutka, Mainpuri, Naswar; ~10 g/day), aged around 60 years, with no other drug use. Group 2 (50 individuals): Healthy, age-matched controls with no history of tobacco or drug use, matched for socioeconomic status, locality, and diet. Blood (3–5 ml) was collected using metal-free Safety Vacutainer tubes, stored in CP bottles, and preserved at 5 °C. All samples were handled with attention to temperature control and contamination prevention^26^. A sociodemographic questionnaire was also administered to each participant, covering personal, occupational, and dietary information.

### The conventional wet acid digestion method (CDM)

Triplicate samples of 1.0 mL blood and 0.5–1.0 g of tobacco products (cigarette, gutka, mainpuri, naswar), along with six replicates of Standard Reference Material (SRM 1575 a, Pine Needles), were placed in 100 mL conical flasks. Each sample was digested using 20–50 mL of concentrated nitric acid (65%) and 2.0–5.0 mL of hydrogen peroxide (30%), then heated at 250 °C for 20–60 min until a clear solution formed. After cooling and dilution with distilled water, the solutions were filtered (Whatman 42 filter paper) and transferred to 10 mL volumetric flasks for analysis. Metal concentrations were determined using ETAAS/FAAS spectrometry. Blanks and spiking recovery tests were processed similarly to validate accuracy. All procedures followed strict ethical guidelines approved by the PCSIR Institutional Review Committee, ensuring safe handling, storage, and transport of blood samples in line with biohazard protocols^27^.

### Statistical analysis

(All experimental data were processed using Minitab 13.2 (Minitab Inc., State College, PA), Minitab, and Origin Pro (9.0) and Microsoft Excel (2013). The results are presented as mean ± standard deviation (SD). Statistical analysis was conducted using one-way ANOVA and t-tests at a 0.05 significance level to evaluate variations in TTE concentrations among different cigarette brands, STPs, and blood samples and control healthy subjects (nonsmokers) residing in urban and rural areas of Karachi.

### Average daily intake (ADI)

The Average Daily Intake (ADI) of potentially TTEs was calculated for all analyzed samples, including cigarettes and STPs like gutka, mainpuri, and naswar. The ADI was determined using the following equation:

Where:

1 $$\:\text{A}\text{D}\text{I}=\:\frac{\text{C}\text{M}\left(\frac{\text{m}\text{g}}{\text{K}\text{g}}\right)\text{x}\:\text{A}\text{C}\:\left(\text{g}\right)\:}{\text{B}\text{W}\:\left(\text{K}\text{g}\right)}$$

Where ADI is Average daily intake (10 to 20 g cigarttes/10 g STPs per day, CM is representing concentration of metal in mg/kg, Average consumption (AC) of intake of cigarette per day approximately for male smoker 20 g cigarette/day by body weight of 70 Kg and for female 10 g cigarettes per day by body weight of 60 Kg respectively, (g/person/d). Similarly for STPs taken 10 g for each products and body weight is smilar as described in cigerttes. Tolerable daily intakes (TDI) of metals^28,29^ were compared with the ADI of studied metals.

### Chronic daily intake (CDI)

The CDI of potentially TTEs was calculated for all analyzed samples, including cigarette and STPs and whole blood of male and female of both areas. The approximate age of tobacco products users is 60 years and the the addiction assumed is 15 years, so the exposure duration would be 45 years. The CDI was determined using the following Eqs^30,31^.:

Where:

2 $$\:\text{C}\text{D}\text{I}=\:\frac{\text{A}\text{D}\text{I}\:\left(\frac{\text{m}\text{g}/\text{K}\text{g}}{\text{d}\text{a}\text{y}}\right)\text{x}\:\text{E}\text{F}\:\text{x}\:\text{E}\text{D}\:}{\text{A}\text{T}\:\text{f}\text{o}\text{r}\frac{\text{C}\text{R}}{\text{A}\text{T}}\text{f}\text{o}\text{r}\:\text{N}\text{C}\text{R}}$$

CDI = Chronic Daily Intake (mg/kg/day), ADI = Average Daily Intake (mg/day), EF = Exposure Frequency = 365 days/year ED = Exposure Duration = years, AT = Averaging Time, For non-carcinogenic risk: 45 years × 365 = 16,425 days, For carcinogenic risk: 70 years × 365 = 25,550 days.

### Estimation of target hazard quotient (THQ)

The THQ for each TTEs, including Cd, Mn, Pb, and Zn, was calculated in order to assess the daily intake of these metals. THQ was calculated as per USEPA Region III Risk-based Concentration Table^32^. THQ was determined using the following formula by dividing the estimated Average Daily Intake (ADI) of each metal from cigarettes and STPs by its respective oral Reference Dose (RfD):

3 $$\:\text{T}\text{H}\text{Q}=\:\frac{\text{A}\text{D}\text{I}\:}{\text{R}\text{f}\text{D}}$$

In this equation, THQ represents the Target Hazard Quotient, ADI (mg/kg/day) is the Average Daily Intake and, The RfD (Refrence Dose) . values, provided by the^33^are as follows: Cd 0.001 mg/kg/day, Mn 0.14 mg/kg/day, Pb 0.0035 mg/kg/day, and Zn 0.30 mg/kg/day as provided by U.S. Environmental Protection Agency, 2023^34^.

### Hazard index (HI)

The Hazard Index (HI) was calculated by summing the THQs of Cd, Mn, Pb, and Zn for cigarette samples and as well as for STPs. The HI for cigarettes and STPs was determined using the respective THQs for each TTE, following the Eq. (4) and the method described by^35^.

4 $$\:\text{H}\text{I}\hspace{0.17em}=\hspace{0.17em}\text{T}\text{H}\text{Q}\:\left(\text{C}\text{d}\right)\hspace{0.17em}+\hspace{0.17em}\text{T}\text{H}\text{Q}\:\left(\text{P}\text{b}\right)\hspace{0.17em}+\hspace{0.17em}\text{T}\text{H}\text{Q}\:\left(\text{M}\text{n}\right)\hspace{0.17em}+\hspace{0.17em}\text{T}\text{H}\text{Q}\:\left(\text{Z}\text{n}\right)$$

The HI > 1 for any commodity means that the health of population/consumer is at risk.

### Estimation of carcinogenic risk (CR)

The carcinogenic risk (CR) associated with exposure to potentially TTEs, including Cd, Mn, Pb, and Zn, CDIs was estimated using the values presented in Table 1 . According to established guidelines, a CR value between 1 × 10⁻⁶ and 1 × 10⁻⁴ is considered acceptable. Non-carcinogenic risk (NCR) and CRwere calculated for each TTE in all tested products using the following Eq. 5:

5 $$\:\text{C}\text{R}=\text{C}\text{D}\text{I}\text{x}\text{C}\text{S}\text{F}$$

Where CR = Carcinogenic Risk, CDI = Chronic Daily Intake (mg/kg/day), CSF = Cancer Slope Factor for Cd (oral): 6.1 (mg/kg/day) and for Pb: 0.0085 (mg/kg/day)^34^, Averaging Time (AT): as described in CDI section. However, Mn and Zn are essential elements and therefore are not associated with carcinogenic risk.

## Results and discussion

A total of 310 samples were collected, including 190 whole blood samples from cigarette and STPs users and 50 from healthy non-smokers. Additionally, 120 samples of various tobacco products (cigarette, gutka, mainpuri, naswar) were collected from the Karachi market. All samples were analyzed for potentially TTEs using nitric acid and hydrogen peroxide digestion, followed by FAAS and ETAAS. The study focused on users around 60 years old and highlighted high tobacco use among adolescents, especially in low-income groups, due to easy access and low cost. Findings were compared with healthy individuals from urban and rural Karachi.

### Concentration of TTEs in cigarette and STPs

The results of TTEs in cigarettes and STPs reveals significant variations. Cadmium (Cd) levels were highest in gutka (1.11 ± 0.21 mg/kg), followed by cigarettes (1.035 ± 0.08 mg/kg), both exceeding the WHO permissible limit of 0.5 mg/kg. Manganese (Mn) showed the highest concentration in cigarettes (49.19 ± 4.11 mg/kg) and mainpuri (39.8 ± 3.26 mg/kg), with comparatively lower levels in gutka (13.8 ± 1.25 mg/kg) and naswar (1.87 ± 0.03 mg/kg). Pb levels were notably elevated in gutka (3.08 ± 0.25 mg/kg) and naswar (2.18 ± 0.17 mg/kg), far exceeding the WHO limit of 0.2 mg/kg. Zinc, while essential in trace amounts, was found in high concentrations in cigarettes (58.7 ± 5.12 mg/kg), well above the WHO permissible limit of 5 mg/kg^37^. Overall, cigarettes and gutka pose the highest toxic metal exposure risk, indicating urgent public health concerns, especially for low-cost, widely accessible STPs (Table 1).

**Table 1:** Average values of Cd, Mn, Pb, and Zn level in Cigarette, Gutka, Mainpuri, and Naswar samples and whole blood samples of Smoker and Non-Smoker, STP user in urban and rural areas. All results are expressed in mg/kg and for blood mg/L.

Metals	Cigarette		Gutka		Mainpuri		Naswar	
Cd	1.035 ± 0.08		1.11 ± 0.21		0.144 ± 0.01		0.547 ± 0.04	
Mn	49.19 ± 4.11		13.8 ± 1.25		39.8 ± 3.26		1.87 ± 0.03	
Pb	0.872 ± 0.07		3.08 ± 0.25		1.01 ± 0.05		2.18 ± 0.17	
Zn	58.7 ± 5.12		19.3 ± 1.76		15.9 ± 1.23		1.89 ± 0.16	
**Cigarette Smokers from Urban Areas**					**Cigarette Smokers from Rural Areas**			
	MS-UA	FS-UA	MNS-UA	FNS-UA	MS-RA	FS-RA	MNS-RA	FNS-RA
Cd	0.055 ± 0.003	0.036 ± 0.001	0.0058 ± 0.0003	0.0048 ± 0.0003	0.041 ± 0.004	0.034 ± 0.001	0.0028 ± 0.0001	0.0024 ± 0.0001
Mn	0.029 ± 0.002	0.031 ± 0.001	0.011 ± 0.001	0.011 ± 0.001	0.019±0.001	0.019 ± 0.001	0.0057 ± 0.0003	0.0089 ± 0.0006
Pb	0.108 ± 0.01	0.085 ± 0.004	0.007 ± 0.0005	0.003 ± 0.0001	0.059 ± 0.004	0.051 ± 0.004	0.003 ± 0.0001	0.003 ± 0.0002
Zn	0.631 ± 0.04	0.623 ± 0.03	0.596 ± 0.04	0.516 ± 0.05	0.623 ± 0.04	0.636 ± 0.04	0.532 ± 0.03	0.525 ± 0.04
**Smokeless Tobacco Products user from Urban Areas**								
		MG-UA	FG-UA	MMP-UA	FMP-UA	MN-UA	FN-UA	
Cd		0.012 ± 0.001	0.014 ± 0.001	0.013 ± 0.001	0.012 ± 0.001	0.011 ± 0.001	0.008 ± 0.0001	
Mn		0.014 ± 0.001	0.013 ± 0.001	0.015 ± 0.001	0.014 ± 0.001	0.016 ± 0.001	0.015 ± 0.001	
Pb		0.028 ± 0.001	0.026 ± 0.001	0.027 ± 0.001	0.025 ± 0.001	0.025 ± 0.001	0.023 ± 0.001	
Zn		1.42 ± 0.12	1.23 ± 0.11	1.33 ± 0.12	1.14 ± 0.13	1.38 ± 0.13	1.23 ± 0.13	
**Smokeless Tobacco Products user from Rural Areas**								
		MG-RA	FG-RA	MMP-RA	FMP-RA	MN-RA	FN-RA	
Cd		0.010 ± 0.001	0.009 ± 0.001	0.007 ± 0.001	0.008 ± 0.001	0.009 ± 0.0006	0.008 ± 0.0007	
Mn		0.014 ± 0.001	0.012 ± 0.001	0.009 ± 0.0007	0.013 ± 0.001	0.015 ± 0.001	0.013 ± 0.001	
Pb		0.018 ± 0.001	0.017 ± 0.001	0.010 ± 0.001	0.014 ± 0.001	0.016 ± 0.001	0.014 ± 0.001	
Zn		1.33±0.13	1.16±0.11	2.76 ± 0.16	1.11 ± 0.11	1.30 ± 0.13	1.14 ± 0.11	

The % relative standard deviation (RSD) for all measured concentrations was less than 10%.

### Metals in blood of cigartee and STPs user

Tobacco use is a growing health concern due to toxic elements like Cd, Mn, Pb, and Zn, which pose carcinogenic and neurotoxic risks^38^. This study analyzed TTEs levels in various cigarette and STPs users blood samples collected from urban and rural areas.

### Cadmium (Cd)

**Fig. 1:**
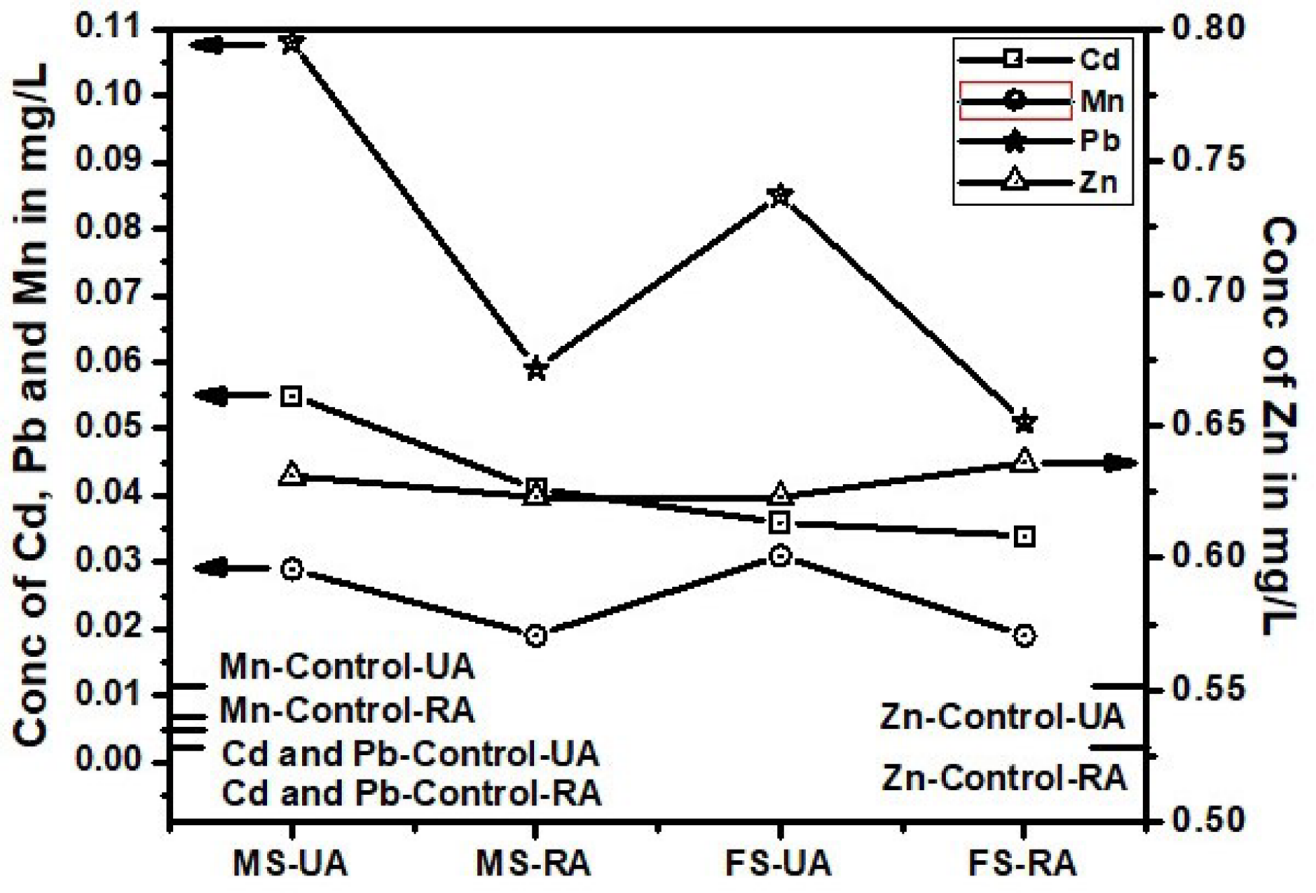
Trace toxic metals in blood of cigarette smokers from urban and rural areas.

**Fig. 2:**
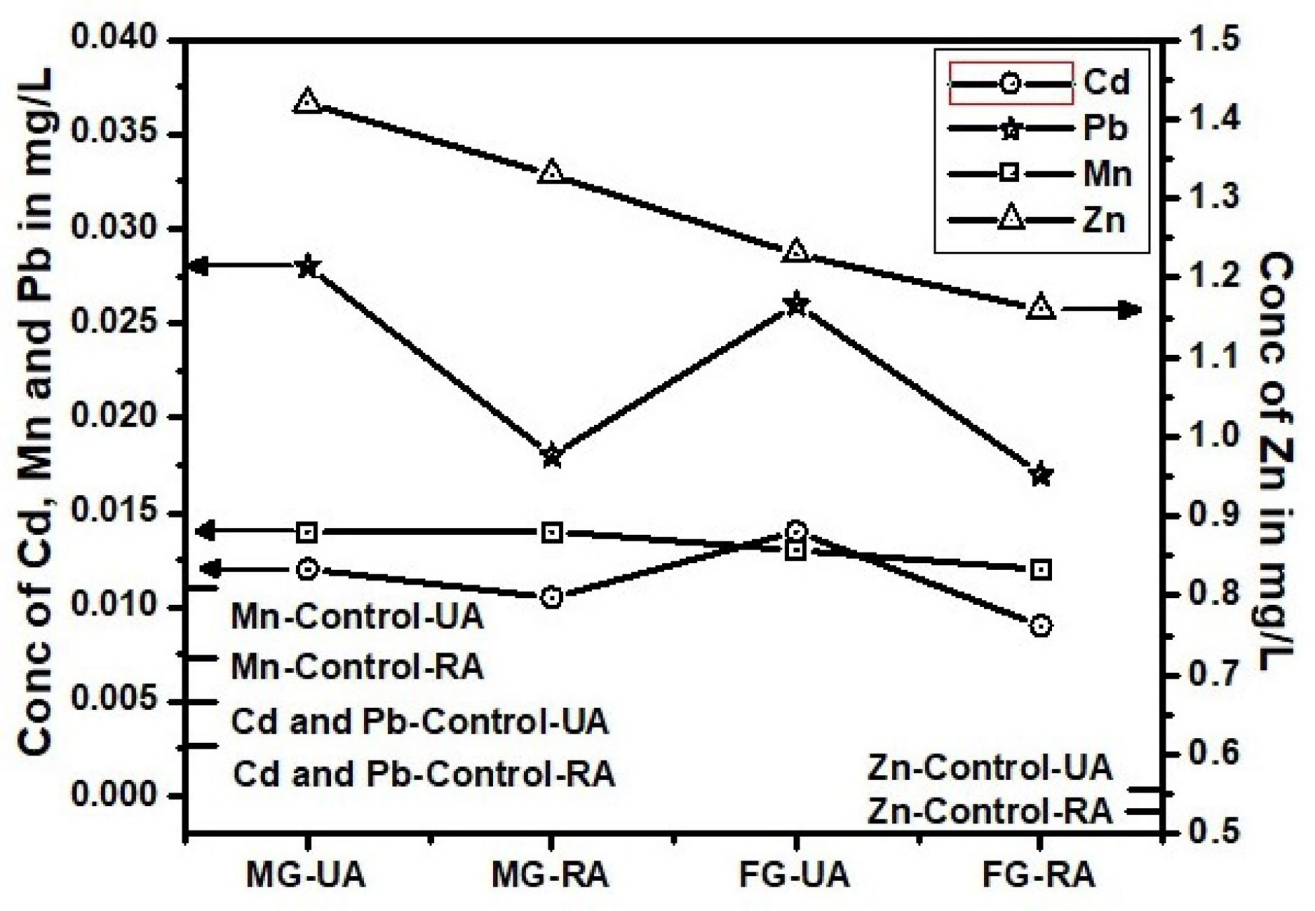
Trace toxic metals in blood of gutka users from urban and rural areas.

**Fig. 3:**
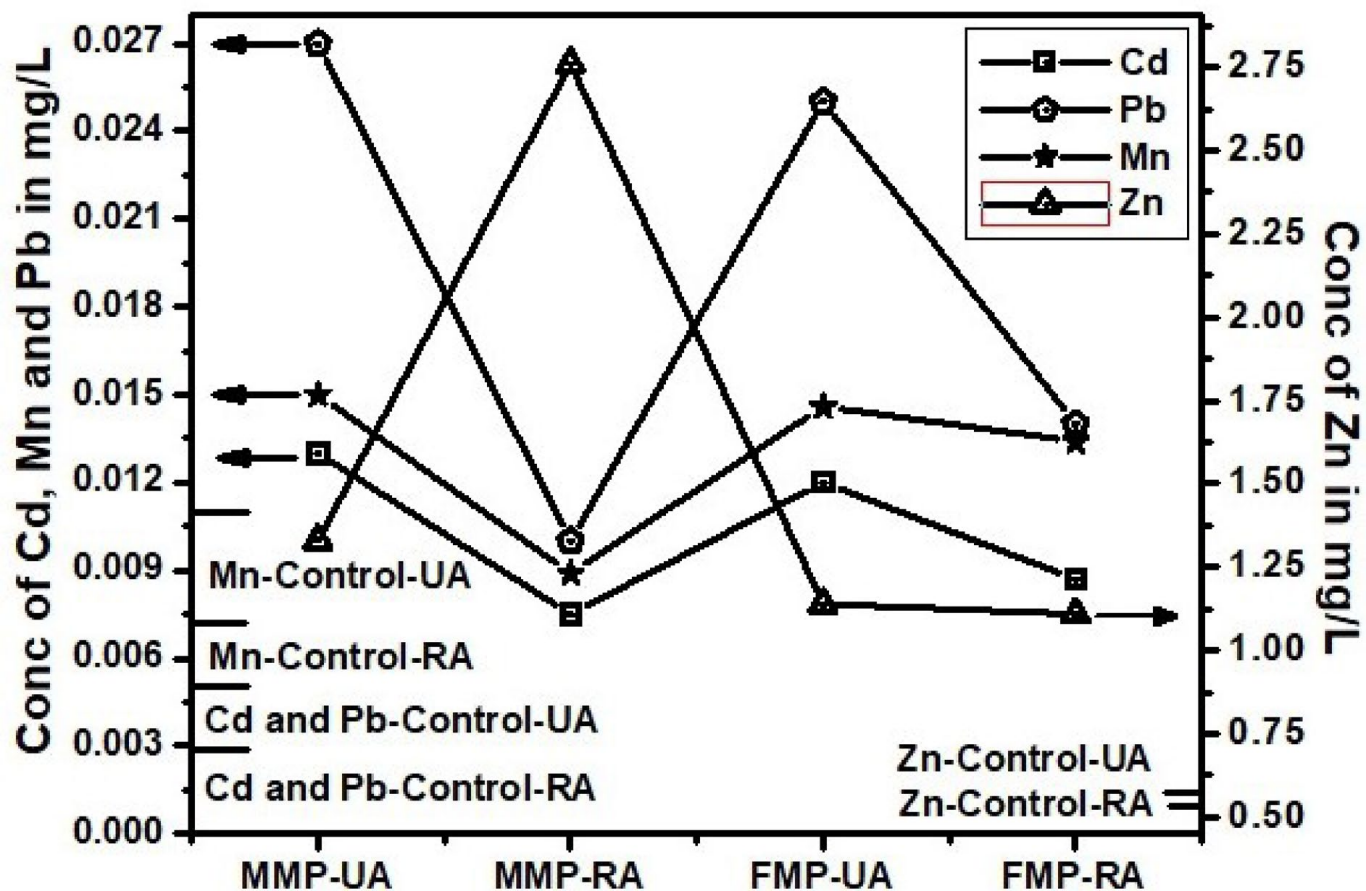
Trace toxic metals in blood of Mainpuri users from urban and rural areas.

**Fig. 4:**
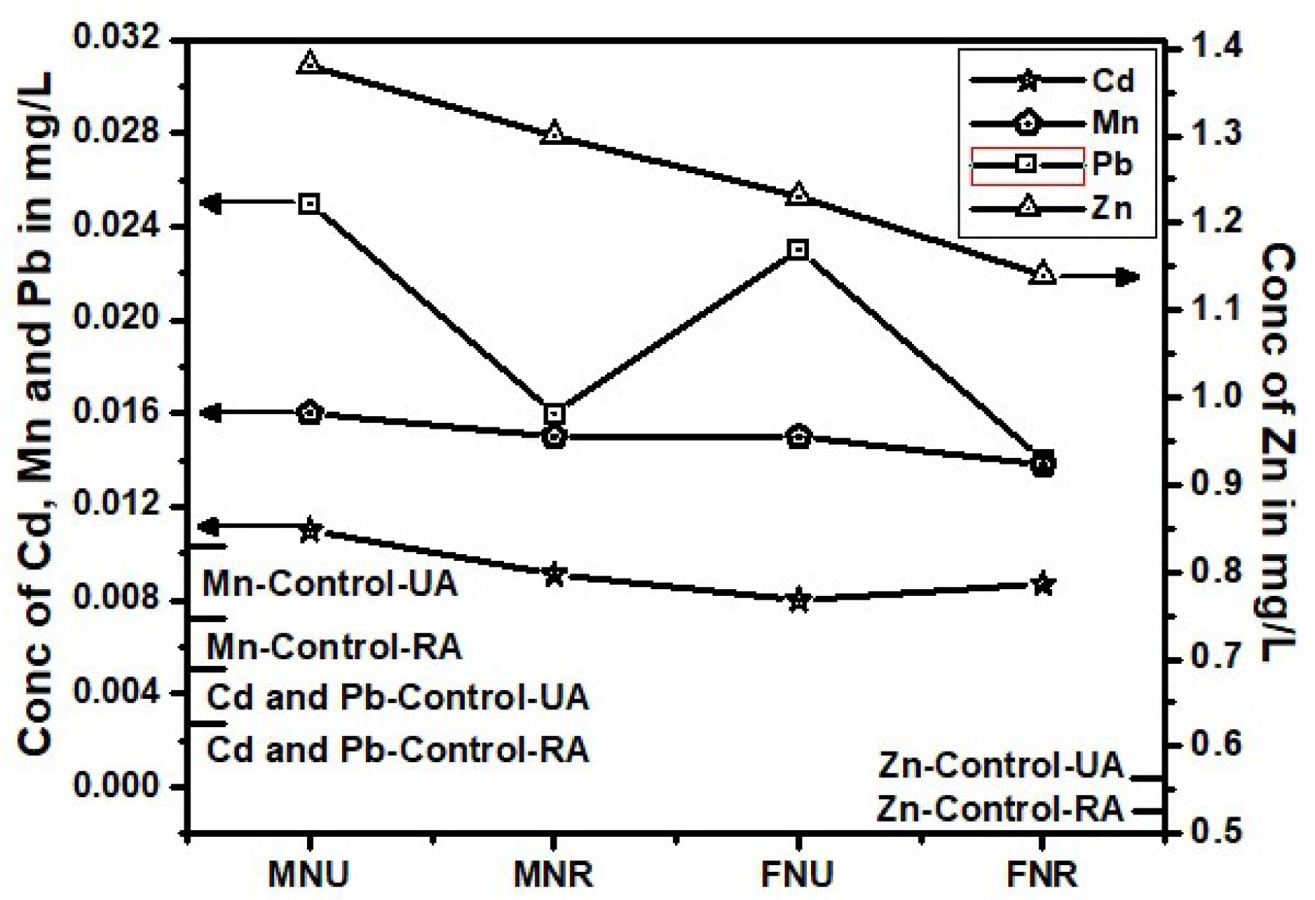
Trace toxic metals in blood of Naswar users from urban and rural areas.

Cadmium (Cd) exposure is significantly linked to tobacco use, as evidenced by markedly higher blood Cd levels in smokers compared to non-smokers. Urban male smokers (0.055 ± 0.003 mg/L) show nearly tenfold higher Cd concentrations than non-smokers (0.0058 ± 0.16 mg/L), clearly indicating that smoking is a major source of Cd exposure (*p* < 0.05), Table 1. Female smokers also exhibit elevated levels, although slightly lower than males. This trend persists across rural populations at slightly reduced concentrations, suggesting that environmental and lifestyle differences influence exposure. Moreover, STPs such as gutka, mainpuri, and naswar contribute significantly to Cd accumulation, especially among urban users. Urban male users of mainpuri had the highest Cd levels (0.013 ± 0.001 mg/L) (Table-1). The trend of Cd in both areas and four products types remain almost similar, below other three metals and above control values (Figs. 2, 3 and 4). Except, the higher accumulation was observed than Mn in cigarette smokers blood as shown in Fig. 1. These findings supports, the reported studies that smokers have 1–4 times higher Cd levels than non-smokers^39^. Therefore, regulating tobacco use and addressing urban environmental pollution are critical to reducing Cd-related health risks. Variations in Cd levels between urban and rural smokers may result from differences in tobacco type, usage frequency, and added exposure from urban pollution and industry. Urban smokers face higher risks from combined tobacco use and environmental pollution. Elevated Cd levels support global calls for stricter controls, public health measures, and regular health checks for vulnerable groups^40^.

### Manganese (Mn)

Although Mn is essential for enzyme function and antioxidant defense, its excessive accumulation, especially via inhalation, poses serious neurotoxic risks, including links to Parkinson’s and Alzheimer’s diseases^41^. This study presents compelling evidence that tobacco use significantly contributes to elevated blood Mn levels, particularly among urban smokers. Urban female smokers (0.031 ± 0.001 mg/L) showed slightly higher Mn levels than urban males (0.029 mg/L), both exceeding rural smoker values (~ 0.019 mg/L), suggesting an additive effect of tobacco and urban pollution (*p* < 0.05). Non-smokers had the lowest Mn levels (0.011 mg/L), yet urban non-smokers still surpassed rural smokers, underscoring the environmental burden of city living (Table 1). On the contrary, the Mn level remain lower than Cd, which is the most lowest in the blood of STPs users (Figs. 1, 2, 3 and 4). The reason behind higher Mn blood level is higher concentration of Mn (49.19 ± 4.11 mg/kg) in Cigarette samples among all other tobacco samples (Table-1). The Mn level in female remain lower than male blood samples among STPs users for urban and rural both (Figs. 2, 3 and 4).

Across STP users, urban mainpuri and naswar users also showed higher Mn concentrations compared to rural users, particularly among males (*p* < 0.05). The consistent pattern of elevated Mn among urban users reflects exposure from tobacco, diet, and industrial pollution. As tobacco plants accumulate Mn and combustion leads to inhalation exposure, this aligns with prior studies^42^.

### Lead (Pb)

Lead (Pb) exposure remains a critical health issue. The WHO safe limit for blood Pb is 0.01 mg/L (10 µg/dL). In this study, urban male smokers (MS-UA) had the highest Pb concentration at 0.108 ± 0.01 mg/L, exceeding the WHO limit over tenfold. Urban female smokers (FS-UA) had 0.085 ± 0.006 mg/L, also well above safe levels. In rural smokers, Pb levels were lower: MS-RA averaged 0.059 ± 0.004 mg/L, and FS-RA had 0.051 ± 0.003 mg/L, with no significant gender difference (*p* > 0.05). Non-smokers in both urban and rural areas showed low Pb levels i.e. MNS-UA: 0.007 ± 0.0005 mg/L and FNS-UA, MNS-RA, FNS-RA: 0.003 ± 0.0002 mg/L. These values fall within safe WHO limits, though they indicate background exposure. For gutka users, urban males lead blood levels were (MG-UA) had 0.028 ± 0.001 mg/L, and females (FG-UA) had 0.026 ± 0.001 mg/L. But Rural levels were lower as for male MG-RA: 0.018 ± 0.001 mg/L, for female FG-RA: 0.017 ± 0.001 mg/L. Similarly Mainpuri and naswar users in urban areas were 0.027 ± 0.001 and 0.025 ± 0.001 mg/L for males and 0.025 ± 0.001 and 0.023 ± 0.001 mg/L for female respectively. The rural area male mainpuri and naswar blood Lead levels were 0.010 ± 0.001 and 0.016 ± 0.001 mg/L respectively. Whereas for female the levels were 0.014 ± 0.001 and 0.023 ± 0.001 mg/L with significant difference by location, *p* < 0.05 respectively (Table-1). Urban STPs users averaged between 0.023 and 0.028 mg/L, while rural users ranged from 0.014 to 0.018 mg/L. Among all STPs, gutka users showed the highest Pb concentrations. Both urban and rural male showed highest Pb level that could be evident from Fig. 1. STPs users blood Pb showed same trend as of cigarette from Figs. 2, 3 and 4.

### Zinc (Zn)

Zinc (Zn), an essential trace element, plays a critical role in enzymatic activity, immune function, and cellular repair. In this study, Zn concentrations in tobacco users varied by product type, gender, and location, though most remained within or slightly above normal physiological limits. Among cigarette smokers, Zn levels were moderately higher than in non-smokers. Urban male smokers (0.631 ± 0.05 mg/L) and rural females (0.636 ± 0.04 mg/L) showed the highest values, while non-smokers exhibited lower levels (e.g., FNS-UA: 0.516 ± 0.05 mg/L). Though these differences were not statistically significant (*p* > 0.05), they suggest smoking-induced oxidative stress may enhance Zn retention^43^.

Smokeless tobacco product (STPs) users, especially gutka and naswar users, had consistently higher Zn levels. Gutka-using urban males recorded 1.42 ± 0.12 mg/L, while naswar-using urban males had 1.38 ± 0.13 mg/L. Females generally showed lower Zn concentrations than males. A striking anomaly was observed among rural male mainpuri users, who had a significantly elevated Zn level of 2.76 ± 0.16 mg/L (*p* < 0.05) the highest among all groups, indicating possible exposure through contaminated ingredients, environmental sources, or local production additives. In contrast, rural female mainpuri users had 1.11 ± 0.11 mg/L. Overall, male users had higher Zn levels than females, and urban users slightly more than rural ones, except for rural male mainpuri users. The elevated Zn in STP users may reflect oxidative stress-related retention and unregulated product composition^44^. Although Zn is less toxic than Cd or Pb , chronic excess poses health risks, underscoring the need for regular biomonitoring and regulation of STPs^45^.

### Analytical figures of merit

The proposed method for analyzing trace metals (Cd, Pb, Mn, and Zn) was rigorously validated through repeatability, linearity, and recovery tests. Calibration curves demonstrated excellent linearity with correlation coefficients of 0.9995 for Cd, 0.9996 for Mn, 0.9997 for Pb, and 0.9999 for Zn over the ranges of 1–5 µg/L for Cd and Mn, 10–50 µg/L for Pb, and 0.5–1.5 mg/L for Zn. The limits of detection (LOD) were 0.003 mg/L for Cd, 0.025 mg/L for Pb, 0.01 mg/L for Mn and Zn, while limits of quantification (LOQ) were calculated as 10 times the standard deviation of blanks. Relative standard deviations (RSDs) for triplicate analyses were all under 10%, confirming good precision.

### Accuracy of the method

The proposed methodology was validated using Standard Reference Material (SRM 1575a: Pine Needles, *Pinus taeda*) and a spiking recovery test. Standard Reference Material (SRM 1575a) showed high recovery rates: 98.3% (Cd), 97.3% (Mn), 98.8% (Pb), and 99.5% (Zn). Spiking recovery tests yielded similar results: 98.1% (Cd), 99.7% (Mn), 97.3% (Pb), and 99.6% (Zn). These results matched certified reference values at a 95% confidence level, confirming method accuracy and reliability (Table 2). Robust analytical signals and consistent recoveries were observed across multiple concentration levels (e.g., 1.0 µg/L for Cd/Mn, 20.0 µg/L for Pb, and 1.0/15.0 mg/L for Zn) using ETAAS. Statistical tests confirmed that all results were significant at *p* < 0.05, supporting the method’s precision, linearity, and suitability for trace metal analysis in real biological samples.

**Table 2:** Standard reference material **(**Pine needles (Pinus taeda) SRM 1575a) and spiking recovery test results for urban areas of non-smokers in mg/kg.

Analyte	Assigned values	Determined Values	% Recovery	Added Value	Found values	% Recovery
Standard Reference Material				Spiking recovery test in cigarette sample,		
Cd	0.233 ± 0.004	0.229 ± 0.018	98.3	0.0	1.15 ± 0.11	……
				1.0	2.11 ± 0.21	98.1
Mn	488 ± 12	475 ± 39.2	97.3	0.0	56.3 ± 4.67	……
				20.0	76.1 ± 5.15	99.7
Pb	0.167 ± 0.015	0.165 ± 0.01	98.8	0.0	0.182 ± 0.02	……
				1.0	1.15 ± 0.09	97.3
Zn	38 ± 2	37.8 ± 3.65	99.5	0.0	53.8 ± 4.12	……
				15.0	68.5 ± 4.97	99.6

### Application to real samples

A validated analytical method was applied to blood samples from smokers, STPs users, and non-smokers in both urban and rural areas of Karachi to determine levels of Cd, Mn, Pb, and Zn. Blood samples were collected in CP bottles from healthy individuals who were not drug users. Samples were digested using 65% nitric acid on a sand bath at 250 °C, filtered, and analyzed. Results (presented in Table 1) showed the metal concentration order as: Zn > Cd > Mn > Pb. Urban residents had higher levels of all four metals compared to rural residents. The relative standard deviations were all below 10%, confirming good precision. While Zn levels were comparable in both regions, Cd, Mn, and Pb were significantly higher in urban areas, athough the levels of these metals in rural areas remained within safe limits.

### Average daily intake and health risk Estimation

The Average Daily Intake (ADI) of potentially TTEs (Cd, Mn, Pb, Zn) from cigarettes and STPs; gutka, mainpuri, and naswar, revealed significant variations in exposure levels and associated health risks. Cigarettes posed the highest Cd exposure (0.233 mg/kg/day, ADI), mainly due to inhalation, which offers greater metal bioavailability. Smokers also showed higher average ADIs for Mn (11.1 mg/kg/day), Pb (0.197 mg/kg/day), and Zn (13.3 mg/kg/day), indicating elevated risks for neurotoxicity, cardiovascular issues, and cancer (Table 3). Gutka had the highest total metal concentrations among STPs, with Cd, Pb, and Zn ADIs of 0.172, 0.455, and 2.99 mg/kg/day, respectively (Table 3). surpassing cigarette users in Pb and Zn exposure. This suggests a greater overall toxicological risk from gutka use. Mainpuri showed lower Cd intake (0.029 mg/kg/day) but the highest Mn exposure (6.16 mg/kg/day), emphasizing its potential for causing neurological damage. Pb and Zn ADIs were moderate (Table 3). Naswar had the lowest metal contents and ADIs across all elements, making it comparatively less harmful, though still a source of chronic exposure. Overall, gutka and mainpuri users face greater toxic risks than naswar users, and in some cases even cigarette smokers, due to higher levels of heavy metals. Females may be especially vulnerable due to more efficient metal absorption. These findings align with toxicokinetic models and stress urgent public health interventions^46^.

**Table 3:** Estimated Average Daily Intake (ADI), Chronic Daily Intake (CDI), Target Hazard Quotient (THQ), Carcinogenic Risk (CR) and Hazard Index (HI) results are expressed inmg/kg/day.

Metals	ADI	CDI	THQ	CR	HI
Cigarettes					0.413
Cd					
Non cancer	0.233	2.33 × 10^−4^	0.233	1.42 × 10^−3^	
Cancer risk		1.49 × 10^−4^	0.149	9.14 × 10^−4^	
Mn	11.1	1.11 × 10^−2^	7.93 × 10^−2^	NA	
Pb					
Non cancer	0.197	1.97 × 10^−4^	5.63 × 10^−2^	4.78 × 10^−4^	
Cancer risk		1.26 × 10^−4^	3.6210^−2^	1.07 × 10^−6^	
Zn	13.3	1.33 × 10^−2^	4.43 × 10^−2^	NA	
Gutka user					0.334
Cd					
Non cancer	0.172	1.72 × 10^−4^	0.172	1.05 × 10^−3^	
Cancer risk		1.11 × 10^−4^	0.111	6.74 × 10^−4^	
Mn	2.14	2.14 × 10^−3^	1.53 × 10^−2^	NA	
Pb					
Non cancer	0.477	4.77 × 10^−4^	0.136	4.05 × 10^−6^	
Cancer risk		3.06 × 10^−4^	0.088	2.61 × 10^−6^	
Zn	2.99	2.99 × 10^−3^	9.96 × 10^−3^	NA	
Mainpuri user					0.119
Cd					
Non cancer	0.022	2.20 × 10^−5^	2.20 × 10^−2^	1.34 × 10^−3^	
Cancer risk		1.41 × 10^−5^	1.41 × 10^−2^	8.63 × 10^−5^	
Mn	6.16	6.16 × 10^−3^	4.40 × 10^−2^	NA	
Pb					
Non cancer	0.156	1.56 × 10^−4^	4.46 × 10^−2^	1.32 × 10^−6^	
Cancer risk		1.01 × 10^−4^	0.029	8.52 × 10^−7^	
Zn	2.46	2.46 × 10^−3^	8.20 × 10^−3^	NA	
Naswar user					0.184
Cd					
Non cancer	0.085	8.50 × 10^−5^	0.085	5.18 × 10^−4^	
Cancer risk		5.46 × 10^−5^	5.46 × 10^−2^	3.33 × 10^−4^	
Mn	0.289	2.89 × 10^−4^	2.06 × 10^−3^	NA	
Pb					
Non cancer	0.337	3.37 × 10^−4^	9.63 × 10^−2^	2.86 × 10^−6^	
Cancer risk		2.16 × 10^−4^	6.19 × 10^−2^	1.84 × 10^−6^	
Zn	0.293	2.93 × 10^−4^	9.77 × 10^−4^	NA	
WHO/FAO Tolerable daily intake(mg/kg/day)		Reference			
Cd	0.2-1.0	^51^			
Mn	25.0	^52^			
Pb	5.0	^53^			
Zn	50	^54^			

^a^Average daily intake (ADI) = (Avg conc in × 20 Cig)/70 (mg/kg body weight/day), where 20 g (0.020 kg) weight is consumed daily by a person (weight of person male 70 and female for 60 kg).

^b^Parameters did not differ significantly at (*p* ≤ 0.05) TDI is Tolerable Daily Intake derived by the Centres for Disease Control and Preventions, 2024.

### Health risk Estimation

This study evaluated the concentrations of potentially TTEs Cd, Mn, Pb, and Zn in cigarette smokers from both urban and rural areas of Karachi. In addition to measuring concentrations, the study estimated the ADI, CDI, THQ, and CR for cigarette smokers (CS) and users of STPs such as gutka, mainpuri, and naswar. ADI values were compared against WHO/FAO-TDI limits, based on an average consumption of 10–20 g of cigarette tobacco per day^47^.

For Cd, the CDI was 2.33 × 10⁻⁴ mg/kg/day and the THQ was 0.233, indicating low non-carcinogenic risk. However, the CR was 1.42x10-3, suggesting a potential carcinogenic threat, which is above the U.S. EPA’s acceptable range (10⁻⁶ to 10⁻⁴). Over a 70-year lifetime exposure, the CR increased to 9.14 × 10⁻⁴, still within acceptable limits but raising concerns due to Cd’s classification as a known human carcinogen (Table 3). Mn showed no health risk, with an ADI of 11.1 mg/kg/day, CDI of 1.11 × 10⁻² mg/kg/day, and THQ of 7.93 × 10⁻². Since Mn is an essential trace element and not classified as carcinogenic, no CR was calculated, and its current exposure levels were deemed safe (Table 3)^48^.

For Pb, the ADI was 0.197 mg/kg/day, and the CDI was 1.97 × 10⁻⁴ mg/kg/day, with a THQ of 5.63 × 10⁻². The CR for Pb was 1.07 × 10⁻⁶in the carcinogenic risk category, reflecting a very low yet present cancer risk, consistent with its status as a probable human carcinogen^49^. While the values were within safe limits, the findings still point to a need for vigilance (Table 3). Zn exposure among cigarette users showed no significant health threat, with an ADI of 13.3 mg/kg/day, CDI of 1.33 × 10⁻² mg/kg/day, and a THQ of 4.43 × 10⁻²,all below the threshold of concern. THQ, defined as the ratio of metal intake to its reference dose, indicates potential non-carcinogenic risk when it exceeds 1^50^ (Table 3)^51–54^. As Zn is an essential nutrient with no known carcinogenicity, no CR was calculated. Overall, Cd emerged as the most concerning element due to its cancer risk. Mn and Zn posed no health hazards at current levels, while Pb indicated a minor but notable cancer risk. These results align with existing research and emphasize the need for continued monitoring of metal exposure, especially in urban populations where environmental contamination may heighten health risks.

### Risk assessment in cigarette, gutka, Mainpuri and Naswar

This study employed CDI, THQ, and CR to evaluate non-carcinogenic and carcinogenic risks from adult exposure to TTEs, Cd, Mn, Pb, and Zn, through the use of cigarettes, gutka, mainpuri, and naswar. In gutka, Cd posed the most notable carcinogenic risk, with a CR value of 6.74 × 10⁻⁴. Non-carcinogenic metrics for Cd included a THQ of 0.172 and a CDI of 1.72 × 10⁻⁴ mg/kg/day. In gutka, Pb had lower risk levels, with a CR of 2.61 × 10⁻⁶ and a THQ of 0.088. For Mn and Zn, both considered essential elements, no CR was calculated. Their THQs remained well below 1, indicating no significant non-cancer risk (Table 3). Comparable analyses for mainpuri and naswar also showed low THQ and CR values, consistent with minimal health risks under current exposure levels. To assess overall non-carcinogenic risk, Hazard Index (HI) values were calculated. These were 0.413 for cigarette tobacco, 0.334 for gutka, 0.119 for mainpuri, and 0.184 for naswar, well below the critical threshold of 1, suggesting cumulative exposures remain within safe limits (Table 3). Overall, while individual and combined exposures to TTEs via smoking and smokeless tobacco products are within international safety thresholds, the results underscore the contribution of tobacco use to long-term metal exposure in adults. Continued monitoring is essential to mitigate potential health impacts.

### Comparison of TTEs with reported study

#### Comparison in tobacco products

Cadmium (Cd) levels in Karachi cigarette samples in the present study (1.035 ± 0.081 mg/kg) exceeded previously reported values of 0.53 mg/kg in Pakistan^56^. Concentration of Mn (49.19 ± 4.11 mg/kg) in the present work was slightly below the reported 55.4 mg/kg^55^. However, Zn levels (58.7 ± 5.12 mg/kg) in the present study was slightly lower than the reported value 65.0 mg/kg^55^ but significantly higher than 13.39 mg/kg reported in Nigeria^55^. The Cd concentration in Nigerian samples was 0.12 mg/kg^51^. Lead levels reported of 2.08 mg/kg in Pakistan^55^. According to Ajab et al., Cd levels were 0.53 mg/kg, while Pb levels 11.56 mg/kg reported in another Pakistani study^56^. In another study, the authors found that Cd content was slightly below 0.80 mg/kg, while Pb levels were 3.05 mg/kg in cigarette samples from Malaysia^57^ (Table 4).

**Table 4:** Comparison of metal concentration with other reported study for cigarette, gutka, Naswar and Mainpuri products (mg/kg) and in blood (mg/L) levels of users. ,

Sample	Cd	Mn	Pb	Zn	Country Name	References
Cigarette	0.39	55.4	2.08	65.0	Different regions of Pakistan	^55^
	0.53*	---	11.56*	---	Different regions of Pakistan	^56^
	0.80	---	3.05	---	Selangor state, Malaysia	^57^
	0.12	---	2.98	13.39	Samaru Area of Zaria, Nigeria	^51^
Gutka	BDL(< 0.01)	40.27	1.52	27.42	Karachi, Pakistan	^58^
Gutka	0.82	---	3.77	---	Sindh, Pakistan	^59^
Mainpuri	1.65	---	8.66	---		
Naswar (Snuff)	---	0.1674	0.190	0.1674	KPK. Pakistan	^60^
Nass	0.71 (0.25–1.17)	--	38.71 (17. 60–57.70)	--	Iran	^13^
Tobacco snuff	9.7 ± 1.0	--		153–172	Nigeria	^62^
Cigarette	1.035 ± 0.081	49.19 ± 4.11	0.872 ± 0.078	58.7 ± 5.12	Karachi, Pakistan	Present Study
Gutka	1.11 ± 0.21	13.8 ± 1.25	3.08 ± 0.25	19.3 ± 1.76		
Mainpuri	0.144 ± 0.01	39.8 ± 3.26	1.01 ± 0.05	15.9 ± 1.23		
Naswar	0.547 ± 0.04	1.87 ± 0.03	2.18 ± 0.17	1.89 ± 0.16		
**Comparison of metal concentration in blood of Cigarette smokers and Gutka**,** Mainpuri and Naswar users.**						
Cigarette	^**^0.74 ± 0.06 (0.0–2.53)	--	3.83 ± 0.37 (0.4–9.5)	--	**Iran**	^63^
	^*^0.873	--	0.031	--	Al-Najaf, Iraq	^64^
	^*^0.00087	0.0115	0.00638	0.0545	Iran	^65^
	^*^0.00013	0.0125	0.0239	--	Sao Paulo, Brazil	^66^
	^*^0.00091	--	0.02255	--	Lahore, Pakistan	^67^
Gutka	^*^0.00349	--	0.0924	--	Different Regions of Pakistan	^59^
Mainpuri	^*^0.00325	--	0.109	--		
Naswar (Snuff)	^*^> 0.12	--	> 0.1	--	Different Regions of Pakistan	^68^
Cigarette Smoker	^*^0.233	11.1	0.197	13.3	Karachi, Pakistan	Present Work
Gutka	^*^0.172	2.14	0.477	2.99		
Mainpuri	^*^0.022	6.16	0.156	2.46		
Naswar	^*^0.085	0.289	0.337	0.293		

*The values are average of local and imported cigarette.

*mg/L, ^**^µg/dL.

Smokeless tobacco products Gutka contained less than 0.01 mg/kg of Cd, Mnreported value of 40.27 mg/kg, and Pb was 1.52 mg/kg reported in earlier studies^58^. However, Zn in the present study (19.3 ± 1.76 mg/kg) was below the 27.42 mg/kg reported^58^. In contrast, Cd concentrations in STPs such as gutka in the present study was higher, with a level of 1.11 ± 0.21 mg/kg, exceeding the 0.82 mg/kg previously reported. In Mainpuri, Cd (0.144 ± 0.01 mg/kg) in the present study was lower than the reported value of 1.65 mg/kg, while Mn (39.8 ± 3.26 mg/kg) was comparatively high. On the contrary, Pb (1.01 ± 0.05 mg/kg) was well below the 8.66 mg/kg reported^59^. The concentration of Mn (1.87 ± 0.03 mg/kg) in the present work exceeded the reported value of 0.1674 mg/kg, and Pb (2.18 ± 0.17 mg/kg) was also higher than the reported 0.190 mg/kg. Similarly, Zn (1.89 ± 0.16 mg/kg) in the present study exceeded the 0.1674 mg/kg reported^60^. Naswar contained Cd (0.547 ± 0.04 mg/kg) in the present study, which falls within the range of 0.25–1.17 mg/kg as reported^13^. Similarly, Zn (15.9 ± 1.23 mg/kg) in the present work was less than Nigerian snuff (153–172 µg/g) reported^62^. Gutka had the highest Cd and Pb. Mainpuri showed peaks of Mn and cigarettes had elevated Cd and Zn. Naswar, also showed increased contamination (Table 4).

### Heavy metals in blood of tobacco users

Lead (Pb) concentration in the present study (0.197 mg/L) in CSblood was lower than the 3.83 ± 0.37 mg/L reported^63 ^but higher than 0.031 mg/L. However, the Cd level was lower than 0.873 mg/L, as reported^64^. The concentrations of Mn (11.1 mg/L) and Zn (13.3 mg/L) in the present work were the highest when compared to 0.0115 mg/L and 0.0545 mg/L, respectively^65^. The reported study also highlighted Cd level of 0.00091 mg/L, while Pb level 0.02255 mg/L^66^  (Table 1). Cd levels in gutka users (0.172 mg/L) in the present study exceeded the 0.00349 mg/L reported in 2020. Similarly, Cd in cigarette smokers (0.233 mg/L) in the present work was higher than 0.00013 mg/L reported^66 ^and Pb (0.477 mg/L) also exceeded the 0.0924 mg/L^59^. Mn (2.14 mg/L) and Zn (2.99 mg/L) in the present study were moderately elevated. In mainpuri users, Cd (0.022 mg/L) and Pb (0.156 mg/L) in the present work were lower than the reported values of 0.00325 mg/L and 0.109 mg/L, respectively^59^. In naswar users, Cd (0.085 mg/L) and Pb (0.337 mg/L) in the present work were higher than the thresholds of > 0.12 mg/L and > 0.1 mg/L, while Mn (0.289 mg/L) and Zn (0.293 mg/L) were the lowest among all user groups^68^. Cigarette smokers exhibited the highest blood Mn and Zn levels, while gutka users showed elevated Cd and Pb (Table 1).

## Conclusion

This study provides essential baseline data on potentially TTEs such as Cd, Mn, Pb and Zn in the blood of tobacco users from urban and rural Karachi, revealing critical public health concerns. Urban smokers showed significantly higher metal levels due to combined exposure from tobacco, industrial emissions, and unregulated smokeless products like gutka, mainpuri, and naswar. Cigarettes and gutka had the highest Cd concentrations 1.035 mg/kg and 1.11 mg/kg, respectively both exceeding the WHO limit of 0.5 mg/kg. Urban male smokers had the highest blood Pb level (0.108 ± 0.01 mg/L), over ten times the WHO threshold, while urban females had the highest Mn level (0.031 mg/L). Rural male mainpuri users had the highest Zn level (2.76 ± 0.16 mg/L), indicating potential contamination. Risk assessments identified cadmium as the most hazardous, with a lifetime cancer risk of 9.13 × 10⁻⁴. Gutka users had the highest product-specific cancer risk (CR: 6.74 × 10⁻⁴), and females the highest Pb intake (0.513 mg/kg/day). Despite non-carcinogenic risks remaining below critical levels, cumulative exposure necessitates regular monitoring, stricter regulation, and improved agricultural and manufacturing standards, especially in low-regulation settings.

## Data Availability

The authors declare that the data supporting the findings of this study are available within the paper. Should any raw data files be needed in another format they are available from the corresponding author upon reasonable request.
